# MixSIH: a mixture model for single individual haplotyping

**DOI:** 10.1186/1471-2164-14-S2-S5

**Published:** 2013-02-15

**Authors:** Hirotaka Matsumoto, Hisanori Kiryu

**Affiliations:** 1 Department of Computational Biology, Faculty of Frontier Science, The University of Tokyo, 5-1-5 Kashiwanoha, Kashiwa, Chiba 277-8561, Japan

## Abstract

**Background:**

Haplotype information is useful for various genetic analyses, including genome-wide association studies. Determining haplotypes experimentally is difficult and there are several computational approaches that infer haplotypes from genomic data. Among such approaches, single individual haplotyping or haplotype assembly, which infers two haplotypes of an individual from aligned sequence fragments, has been attracting considerable attention. To avoid incorrect results in downstream analyses, it is important not only to assemble haplotypes as long as possible but also to provide means to extract highly reliable haplotype regions. Although there are several efficient algorithms for solving haplotype assembly, there are no efficient method that allow for extracting the regions assembled with high confidence.

**Results:**

We develop a probabilistic model, called MixSIH, for solving the haplotype assembly problem. The model has two mixture components representing two haplotypes. Based on the optimized model, a quality score is defined, which we call the 'minimum connectivity' (MC) score, for each segment in the haplotype assembly. Because existing accuracy measures for haplotype assembly are designed to compare the efficiency between the algorithms and are not suitable for evaluating the quality of the set of partially assembled haplotype segments, we develop an accuracy measure based on the pairwise consistency and evaluate the accuracy on the simulation and real data. By using the MC scores, our algorithm can extract highly accurate haplotype segments. We also show evidence that an existing experimental dataset contains chimeric read fragments derived from different haplotypes, which significantly degrade the quality of assembled haplotypes.

**Conclusions:**

We develop a novel method for solving the haplotype assembly problem. We also define the quality score which is based on our model and indicates the accuracy of the haplotypes segments. In our evaluation, MixSIH has successfully extracted reliable haplotype segments. The C++ source code of MixSIH is available at https://sites.google.com/site/hmatsu1226/software/mixsih.

## Introduction

Human somatic cells are diploid and contain two homologous copies of chromosomes, each of which is derived from either paternal or maternal chromosomes. The two chromosomes differ at a number of loci and the most abundant type of variation is single nucleotide polymorphism (SNP). Most current research does not determine the chromosomal origin of the variations and uses only genotype information for the analyses. However, haplotype information is valuable for genome-wide association studies (GWAS) [1] and for analyzing genetic structures such as linkage disequilibrium, recombination patterns [2], and correlations between variations and diseases [3].

Let us consider a simple example to demonstrate the importance of haplotype information. Suppose that in a gene coding region, there are two SNP loci, each of which has an independent deleterious mutation in either one of the two homologous chromosomes. If both of the two deleterious mutations are located on the same chromosome, the other chromosome can produce normal proteins. On the other hand, if each chromosome contains either one of the two deleterious mutations, the cells cannot produce normal proteins. It is not possible to distinguish these two cases with only genotype information.

There is a group of algorithms for haplotype inference that statistically construct a set of haplotypes from population genotypes [4-8] Review see [9]. These algorithms have been developed in response to technological advances such as SNP arrays that efficiently measure personal genotypes at a genomic scale. The algorithms infer haplotype blocks based on the assumption that the variety of combinations of alleles is very limited. Therefore, these algorithms fail to identify correct haplotypes in regions with low linkage disequilibrium (LD) where there are frequent recombination events. These algorithms also cannot identify spontaneous mutations. These difficulties are partially resolved by using genotypes of pedigrees. However, family data are not always available, and furthermore, they cannot determine the haplotypes of the loci at which all the family members have the same genotype.

**Figure 1:**
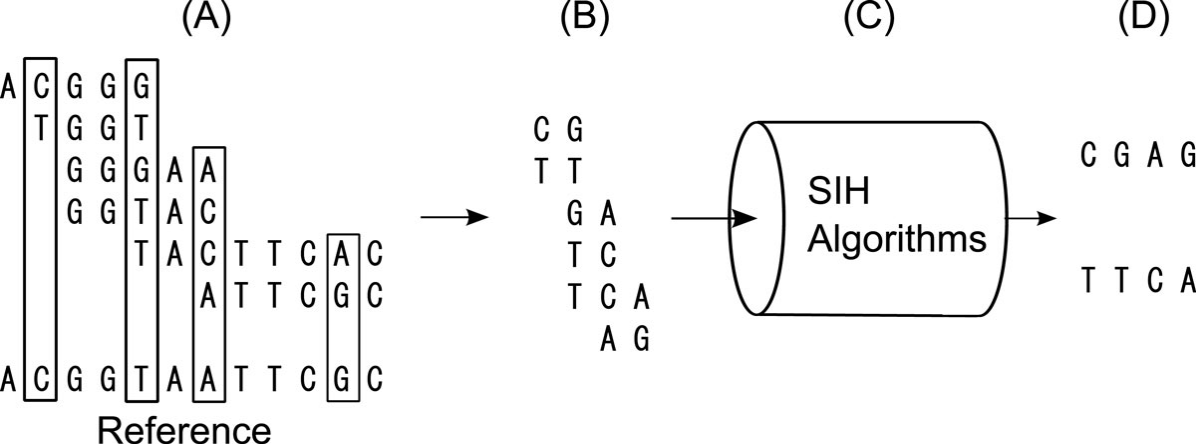
**An illustration of SIH**. An illustration of single individual haplotyping (SIH). The input data for SIH are the SNP fragments (B) which are extracted from the heterozygous alleles in aligned DNA fragments (A). SIH algorithms (C) reconstruct the original haplotypes (D) from the SNP fragments.

Another group of algorithms is single individual haplotyping (SIH) or haplotype assembly. These algorithms infer the two haplotypes of an individual from sequenced DNA fragments [10-17]. These algorithms take as input the read fragments that are aligned to the reference genome, and output the two assembled haplotypes (Figure 1). The algorithms utilize the fact that each read fragment is derived from either one of two chromosomes, though the observed data are a mixture of fragment data from both the chromosomes. If a read fragment spans two or more heterozygous loci, the haplotype can be determined for these sites from the co-occurrence of alleles in the fragment. Two read fragments are determined to originate from the same chromosome if they overlap at a region that has at least one heterozygous locus, and they have the same alleles at these loci. In this case, we obtain a larger haplotype-resolved region by merging the two fragments. The SIH problem is complicated because the fragment data contain many inconsistent fragments caused by sequencing or mapping error.

SIH algorithms did not attract much attention until recently, since the read fragments of next-generation sequencing experiments are not long enough to span multiple heterozygous loci, which exist at only one in one kilo-base on average [18], and the Sanger sequencing that produces long read fragments is too expensive to be conducted at a genomic scale. However, this situation is changing rapidly with the advent of real-time single-molecule sequencing technologies, which are able to sequence DNA fragments as long as 50 kilo-bases [19], and with the development of a novel experimental technique called 'fosmid pool-based next-generation sequencing' [13,20,21], which randomly assigns a bar-code to each read cluster that is derived from the same region in the same chromosome. Because of these advances in experimental techniques, SIH has emerged as one of the most promising approaches for analyzing the haplotype structures of diploid organisms.

The haplotype information which contains errors is likely to lead to wrong results in downstream analyses. For example, in detecting the recombination events from the parent-offspring haplotypes [22], the haplotyping errors are regarded as recombination events by mistake. Another example is that haplotyping errors considerably decrease the detection power of amplified haplotypes in cancer [23] and fetus haplotypes [24]. To use haplotype information in downstream analyses while avoiding such harmful influence of haplotyping errors, it is important not only to assemble haplotypes as long as possible but also to provide means to extract highly reliable haplotype regions. In the statistical haplotype phasing, reliable haplotype regions are determined by selecting the blocks of limited haplotype diversity and level of LD [25-27]. Although there are many algorithms for SIH, none of these algorithms can provide confidence scores to extract reliable haplotype regions.

The algorithms for SIH are classified into two strategies; most of the previous algorithms use deterministic strategies [10-13,15,17] but a few take a probabilistic modeling approach [14,16]. The deterministic algorithms usually include solving the MAX-CUT problem of graph theory [28] in their computational procedures in order to partition the set of the input fragments into two groups representing the two haplotypes. Because these algorithms are designed to optimize only a certain global score function that measures the number of inconsistent fragments and do not model the fragments and haplotypes themselves, it is difficult to produce confidence scores for each region of the assembled haplotypes.

On the other hand, the probabilistic approaches of Kim [14] and Li [16] assume that each observed fragment is sampled from one of the two unobserved haplotypes. Unlike the deterministic approaches, probabilistic models allow the computation of various expected values and confidence values from the Bayesian posterior distributions. For example, Kim [14] and Li [16] defined a confidence value for the haplotype reconstruction of each segment of SNP loci. Unfortunately, those researchers chose a model structure for which the exact computation of the likelihood is extremely computationally intensive. Because the complexity of this summation is exponential in the number of SNP sites, only the posterior probabilities of the haplotypes for neighboring loci are considered. The complete haplotypes are reconstructed by connecting plausible haplotypes of neighboring pairs according to their posterior probabilities. Hence, their approach cannot take into account the full information of fragments that span three or more SNP loci. Their confidence scores for haplotype segments include a summation over all the possible haplotypes, and it is not possible to compute their confidence scores for all the possible segments in the assembled haplotypes.

In this paper, we develop a novel probabilistic SIH model that is very different from the probabilistic models of Kim [14] and Li [16]. Our model takes a 'mixture model' approach: each fragment is emitted completely independently of the other fragments. In contrast, Kim [14] and Li [16] took a 'hidden variables' approach: all the fragments are correlated through hidden haplotype variables (see the Additional file 1 for further explanation). This difference allows us to compute the likelihood with a computational time proportional to the total length of the input fragments. We use the variational Bayes expectation maximization (VBEM) algorithm [29] to compute the approximate posterior distribution of the haplotypes. By using the optimized distribution, we compute the 'minimum connectivity' (MC) score for each segment in the reconstructed haplotypes; this measures whether the segment is free from switch errors. We show that we can extract accurately assembled regions by selecting regions with high MC scores. We also analyze a recent dataset from fosmid pool-based next-generation sequencing and find evidence that the processed dataset contains chimeric fragments derived from the erroneous merging of read clusters in different haplotypes, which degrades the quality of assembled haplotypes significantly.

## Methods

### Algorithms and implementation

#### Notation

Throughout the paper, we denote the number of elements of any set *A *by *|A|*, and the direct product set $\underset{n}{\underset{︸}{A\times \cdots \times A}}$ by *A*^⊗*n*^. Let *X *= {1, 2, . . . , *M*} be the set of SNP loci, and H={0,1} be the two haplotypes. It is convenient to introduce a *phase vector *Φ = *φ*_1 _... *φ_M_*. The pair *φ_j _*= (*φ_j0_*, *φ_j_*_1_) is referred to as *phase*, and represents the two alleles of haplotype 0 and 1 at site *j*, respectively. Because the haplotype assembly problem is trivial for homozygous sites, and because it is usually much easier to determine the genotype than to determine the haplotypes, it is often convenient to restrict the SNP loci *X *to heterozygous sites. Furthermore, if sequence-specific sequencing errors are not considered, it is convenient to use a simple binary representation of alleles; we randomly assign 0 to one of the two alleles at each heterozygous site *j*, and 1 to the other allele. In this case, the set of alleles is denoted by Σ = {0, 1}, and the set of possible phases is denoted by Δ = {(0, 1), (1, 0)}. We assume this binary representation throughout the paper.

Let *F *= {*f_i_|i *= 1, . . . , *N*} be the set of input fragments which are supposed to be aligned to the reference genome, and each fragment *f_i _*takes value *f_ij _*∈ Σ at locus *j *∈ *X *if a nucleotide is aligned and equal to one of two alleles, and *f_ij _*= ∅ if fragment *f_i _*is unaligned, gapped, ambiguous, or a base different from the two alleles, at site *j*. For any subset *X*' ⊆ *X*, we say fragment *f_i _spans *the sites *X' *if *f_ij _*≠ ∅ for all *j *∈ *X'*. We refer to the subset of *X *spanned by fragment *f *as *X*(*f*). We say fragment *f_i _covers *site *j *if there exists a pair of spanning two different (possible non consecutive) SNP sites *j*_1_, *j*_2 _∈ *X*(*f_i_*) such that *j*_1 _*< j ≤ j*_2_. The set of fragments that cover site *j *is denoted by *F^c^*(*j*). Further, we refer to the set of all the possible haplotypes for sites *X*(*f_i_*) as $\Delta \left(f_{i}\right)=\Delta^{⊗|X\left(f_{i}\right)|}$.

The SIH problem takes a set of aligned SNP fragments *F *as input and outputs a hidden phase vector Φ (Figure 1). Because the SIH problem does not associate the inferred haplotypes with the real paternal and maternal chromosomes, the switched configuration $\bar{\Phi }={\bar{\varphi }}_{1}\cdots {\bar{\varphi }}_{M},\;\;{\bar{\varphi }}_{j}=\left({\bar{\varphi }}_{j\bar{0}},{\bar{\varphi }}_{j\bar{1}}\right)$ with $\bar{0}=1$ and $\bar{1}=0$, must be regarded as a completely equivalent prediction. Therefore, SIH has no meaning if there is only one heterozygous site, and it is only meaningful if one considers co-occurrences of alleles on the same haplotype for two or more heterozygous sites.

#### Mixture model

We model the probabilistic distribution of the observed fragments *F *by

$$P\left(F|\Theta \right)=\underset{H\in H^{⊗N}}{\sum }\overset{N}{\underset{i=1}{\prod }}\underset{\Phi^{\left(i\right)}\in \Delta \left(f_{i}\right)}{\sum }P\left(f_{i}|h_{i},\;\Phi^{\left(i\right)}\right)p^{m}\left(h_{i}\right)P\left(\Phi^{\left(i\right)}\right),$$

$$P\left(\Phi^{\left(i\right)}\right)=\underset{j\in X\left(f_{i}\right)}{\prod }p_{j}^{\Phi }\left(\varphi_{j}^{\left(i\right)}\right),$$

where Θ represents a set of parameters defined later, Φ^(*i*) ^∈ Δ(*f_i_*) represents a partial haplotype reconstruction over the sites *X*(*f_i_*) spanned by fragment *f_i_*, *H *= *h*_1 _. . . *h_N _*where $h_{i}\in H$ represents the haplotype origin of fragment *f_i_*, *p^m^*(*h*) is the mixture probability of haplotype $h_{i}\in H$, and $p_{j}^{\Phi }\left(\nu \right)$ is the probability that phase *ν *∈ Δ is instantiated at site *j*. We define the probability of emitting fragment *f_i _*from haplotype *h_i _*given a fixed phase vector Φ^(*i*) ^as follows.

$$P\left(f_{i}|h_{i},\;\Phi^{\left(i\right)}\right)=\underset{j\in X\left(f_{i}\right)}{\prod }p^{e}\left(f_{ij}|\varphi_{jh_{i}}^{\left(i\right)}\right)$$

where,

$$p^{e}\left(\sigma |\sigma^{\prime }\right)=\left\{\begin{array}{cc} \left(1-\alpha \right) & \text{for}\;\sigma =\sigma^{\prime }\\ \alpha & \text{for}\;\sigma \neq \sigma^{\prime } \end{array}\right)$$

is the probability that we observe *σ *∈ Σ when the true allele is *σ' *∈ Σ and *α *represents the sequence error rate which we assume is independent of fragments and positions.

We take *α *as a fixed constant because it is better estimated from other resources rather than from only the bases at the SNP sites. For example, we may estimate *α *by using the all the read sequences or by using information from other dedicated studies about sequencing and mapping errors. In the following, we use *α *= 0.1 unless otherwise mentioned and the dependency of the *α *is described in Additional file 1. We further assume the mixture probabilities are equal, *p^m^*(0) = *p^m^*(1) = 0.5, as they often converge to around 0.5. Therefore, the parameter set Θ that needs to be optimized consists only of the set of phase probabilities: $\Theta =\left\{\theta_{j\nu }\right\}=\left\{p_{j}^{\Phi }\left(\nu \right)\right\}$.

Let $I_{ihj\nu }$ be the indicator function that is one if fragment *f_i _*is derived from haplotype *h*, *X*(*f_i_*) includes *j*, and the haplotypes have phase *ν *at site *j*, and that is zero otherwise. $I_{ihj\nu }$ is uniquely determined if the haplotype origins *H *= {*h_i_|i *= 1, . . . , *N*} and phase vectors Ψ = {Φ^(*i*)^*|i *= 1, . . . , *N*} of fragments *F *are specified. Then the marginalized likelihood *P *(*F|*Θ) is given by

$$\begin{array}{c} P\left(F|\Theta \right)=\underset{H,\Psi }{\sum }P\left(F,\;H,\;\Psi |\Theta \right),\\ \text{log}\left(P\left(F,\;H,\;\Psi |\Theta \right)\right)=N\text{log}\left(0.5\right)+\\ \overset{N}{\underset{i=1}{\sum }}\underset{h\in H}{\sum }\underset{j\in X\left(f_{i}\right)}{\sum }\underset{\nu \in \Delta }{\sum }I_{ihj\nu }\left[\mu_{ihj\nu }+\text{log}\theta_{j\nu }\right],\\ \mu_{ihj\nu }=\text{log}\left(p^{e}\left(f_{ij}|\nu_{h}\right)\right). \end{array}$$

We explain the difference between our model and the models of Kim [14] and Li [16] in Additional file 1.

#### The minimum connectivity score

As described above, the two haplotypes  in the SIH problem have no particular identity and it is not possible to predict which of them converges to the actual paternal or maternal chromosome. In relation to this, the likelihood function *P *(*F*, *H*, Ψ*|*Θ) has a symmetry between the switched configurations: $P\left(F,\bar{H},\bar{\Psi }|\bar{\Theta }\right)=P\left(F,H,\Psi |\Theta \right)$, where $\bar{H}=\left\{{\bar{h}}_{i}|i=1,\ldots ,N\right)$ and $\bar{\Psi }=\left\{{\bar{\Phi }}^{\left(i\right)}|i=1,\ldots ,N\right\}$ represent the configuration that all the haplotype origins of the fragments are exchanged, and $\bar{\Theta }=\left\{{\bar{\theta }}_{jv}\right\},{\bar{\theta }}_{jv}=\theta_{j\bar{v}}$ are the switched phase probabilities. Therefore, the marginal likelihood $P\left(F|\Theta \right)=\sum_{H,\Psi }P\left(F,H,\Psi |\Theta \right)$ is symmetric for the two parameter sets: $P\left(F|\bar{\Theta }\right)=P\left(F|\Theta \right)$.

Suppose that the probabilistic model is optimized for two segments of SNP sites between which there are no connecting fragments, then the association of the haplotypes {0, 1} to the true paternal and maternal chromosomes are selected at random for each segment. Even if there are several connecting fragments, the associations in each segment are determined almost randomly if the number of connecting fragments is not sufficient or there are many conflicting fragments. Such sites often cause switch errors. We define the connectivity at site *j*_0 _as a log ratio of the marginal log likelihoods:

$$\mathrm{connectivity}\left(j_{0}\right)=\text{log}\left(\frac{P\left(F|\Theta \right)}{P\left(F|\Theta^{\prime }\right)}\right)=\text{log}\left(\frac{P\left(F^{c}\left(j_{0}\right)|\Theta \right)}{P\left(F^{c}\left(j_{0}\right)|\Theta^{\prime }\right)}\right)$$

Where $\Theta^{\prime }=\left\{\theta_{jv}^{\prime }\right\}$ with $\theta_{jv}^{\prime }=\theta_{jv}$ for *j *<*j*_0 _and $\theta_{jv}^{\prime }={\bar{\theta }}_{jv}$ for *j *≥ *j*_0_. The second equality follows from the symmetry of *P *(*F|*Θ) described above, and shows that only the fragments covering site *j*_0 _are necessary to compute the connectivity of site *j*_0_. The connectivity measures the resilience of the assembly result against swapping the two haplotypes 0 and 1 in the right part *j *= *j*_0_, . . . , *M *of the sites. We refer to this change of parameters Θ *→ *Θ*' *as *twisting the parameters at site j*_0_.

For each pair of sites (*j*_1_, *j*_2_) (*j*_1 _*< j*_2_), we define the minimum connectivity (MC) score as

$$\text{MC}\left(j_{1},\;j_{2}\right)=\underset{j_{1}<j\leq j_{2}}{\text{min}}\text{connectivity}\left(j\right).$$

We extract confidently assembled regions by selecting the pairs (*j*_1_, *j*_2_) with high MC values. From the above definition, it is obvious that if the MC value is higher than a given threshold for some pair (*j*_1_, *j*_2_), then all the pairs inside range [*j*_1_, * j*_2_] have MC values higher than the threshold. In this sense, MC(*j*_1_, *j*_2_) can be considered as defined on the range [*j*_1_, * j*_2_].

#### Variational bayesian inference

We use the VBEM algorithm to optimize the parameters Θ [29]. We approximate the Bayesian posterior distribution *P *(*H*, Ψ, Θ*|F*) with factorized variational functions *Q*(*H*, Ψ, Θ) = *Q^H^*^Ψ^(*H*, Ψ) ·*Q*^Θ^(Θ) such that the Kullback-Leibler divergence *KL_H_*_ΨΘ_(*Q*(*H*, Ψ, Θ)*||P *(*H*, Ψ, Θ*|F*)) between the two distributions is minimized. The solution to this optimization problem has the form

$$\begin{array}{c} Q^{H\Psi }\left(H,\;\Psi \right)=\frac{1}{Z^{H\Psi }}\text{exp}\left(\overset{N}{\underset{i=1}{\sum }}\underset{h\in H}{\sum }\underset{j\in X\left(f_{i}\right)}{\sum }\underset{v\in \Delta }{\sum }I_{ihjv}\text{log}\left(\beta_{ihj\nu }\right)\right),\\ Q^{\Theta }\left(\Theta \right)=\overset{M}{\underset{j=1}{\prod }}\text{Dir}\left(\theta_{j}|\lambda_{j}\right), \end{array}$$

where *Z^H^*^Ψ ^is a normalization constant, *β_ihjν _*and *λ_jν _*represent the hyperparameters that specify the posterior distributions, and Dir(*θ_j_|λ_j_*) is the Dirichlet probability distribution of *|*Δ*| *parameters. Because *Q^H^*^Ψ^(*H*, Ψ) and *Q*^Θ^(Θ) are connected through the dependencies among the hyperparameters, they cannot be found simultaneously. Therefore, we optimize *β_ihjν _*and *λ_jν _*by an iterative method.

In our model, the parameters often converge to sub-optimal solutions, because switch errors existing in the sub-optimal configurations are not removed by gradual parameter changes. Therefore, we apply a heuristic procedure that re-runs the VBEM several times with twisted parameter configurations after every convergence:

1. Do VBEM and calculate the connectivities for all the sites.

2. Do another VBEM with a parameter set Λ that is twisted at a site with low connectivity.

3. Repeat until convergence.

Here, the twist of hyperparameters Λ = {*λ_jν_*} is defined similarly to that of parameters Θ = {*θ_jν_*}. We describe the details of this procedure in Additional file 1.

#### Inferring haplotypes

We set $p_{j}^{\Phi }\left(v\right)$ to the posterior mean estimate of *θ_jv _*with respect to the converged posterior distribution:

$$p_{j}^{\Phi }\left(v\right)=\int d\Theta \theta_{jv}Q_{\Theta }\left(\Theta \right)=\frac{\lambda_{jv}}{\sum_{v^{\prime }}\lambda_{jv^{\prime }}}.$$

We select the phase *ν *at site *j *for which this $p_{j}^{\Phi }\left(v\right)$ is the highest. We limit the predicted haplotype segments to the regions with high MC values.

#### Possible extensions of the model

In this paper, we consider only the binary representation of heterozygous sites. We also constrain the error rate to be constant throughout the sequence. However, some of these constraints are easily removed. We can include homozygous sites and four nucleotide alleles by expanding the phase set Δ. For example, the phase set of a multi-allelic variant is represented like Δ = {(A,C),(A,G),(C,A),(C,G),(G,A),(G,C)}. We can even include small structural variations if they can be represented by additional allele symbols and the phase set of a structural variant is represented such as Δ_1 _= {(A,-),(-,A)} for indel and Δ_2 _= {("AC","ACAC"),("ACAC","AC")} for short tandem repeats. With these extensions, the accuracy of genotype calling of multi-allelic variants from sequencing data might be improved by considering haplotypes simultaneously [30] and the accuracy and the recall of the haplotype region might be improved because all variant sites add information to infer the derivation of the fragments. Furthermore, we can make the error probability matrix *p^e^*(*σ|σ'*) dependent on the alleles of each fragment, which may be useful for incorporating the quality scores of sequenced reads.

## Datasets and data processing

### Dataset generation

Simulation data were created through a strategy similar to the one reported by Geraci [31]. We first generated *M *binary heterozygous phase vectors and then we generated SNP fragments by replicating each haplotype *c *times and randomly dividing them into subsequences of length between *l*_1 _and *l*_2_. We then randomly flipped the binary values of the fragments from 0(1) to 1(0) with probability *e*. In the following, we use *M *= 1000, *c *= 5, *l*_1 _= 3, *l*_2 _= 7 and *e *= 0.1 unless otherwise mentioned.

For the real data, we used the dataset of Duitama's work [13], who conducted fosmid pool-based next-generation sequencing for HapMap trio child NA12878 from the CEU population. NA12878 had about 1.65 × 10^6 ^heterozygous sites on autosomal chromosome and the haplotypes of about 1.36*×*10^6 ^sites were determined by a trio-based statistical phasing method [18]. In the fosmid pool-based next-generation sequencing, the diploid genomic DNA was fragmented into pieces of length about 40 kilo-bases, and partitioned into 32 pools with low concentration, so that the fragments were long enough to span several heterozygous sites and each pool rarely contained homologous chromosomal regions of different haplotypes. Each pool was sequenced separately using a next-generation sequencer and the read data were mapped onto the reference genome. Since a read cluster in which the reads were close to each other and had the same pool origin were supposed to originate from the same DNA fragment, the alleles observed in the same cluster were merged into a SNP fragment. Duitama [13] converted the fragment data to a binary representation by collecting only the alleles of the heterozygous sites determined by the 1000 genomes project. The coverage of the data was about 3.03. We used the trio-based data and the sequencing data in binary format for our experiment.

The normalized linkage disequilibrium *D' *for the CEU population was downloaded from the HapMap Project [2].

We compared our MixSIH software with ReFHap [13], FastHare [17], DGS [15], which were implemented by Duitama [13], and HapCUT [11]. We selected these algorithms because they have been shown to be superior to other algorithms [13].

For the comparison of the runtimes, we generated simulation data with *M *= 100, 200, 500, 1000. We repeated the measurement 10 times for each *M *and the average runtimes are reported here. The computations were performed on a cluster of Linux machines equipped with dual Xeon X5550 processors and 24 GB RAM.

### Accuracy measures

As described in the introduction, our algorithm is focusing on extracting the reliable haplotype regions. To examine whether we have succeeded in extracting the reliable haplotype regions, an accuracy measure which evaluates the quality of the piecewise haplotype regions is needed. However, existing accuracy measures are designed to compare the efficiency between the algorithms and are not suitable for evaluating the quality of the piecewise haplotype regions.

Let Φ^(*t*) ^be the true haplotypes, and Φ be inferred haplotypes. Because the inferred haplotypes Φ are sets of partially assembled haplotype segments Φ = (Φ_1_, Φ_2_, * . . . *, Φ*_B_*) where each of Φ*_b _*is independently predicted, the accuracy measures have to be applicable for such predictions.

Many previous papers used the Hamming distance to measure the quality of assembled haplotypes [31]:

$$\begin{array}{c} R\left(\Phi_{0}\right)=1-\frac{1}{2M}\text{min}\left[D\left(\Phi_{0},\;\Phi^{\left(t\right)}\right),\;D\left(\Phi_{0},{\bar{\Phi }}^{\left(t\right)}\right)\right]\;,\\ D\left(\Phi ,\;\Phi^{\prime }\right)=\overset{M}{\underset{j=1}{\sum }}\underset{h\in H}{\sum }I\left(\varphi_{jh}={\varphi^{\prime }}_{jh}\right), \end{array}$$

where Φ_0 _represents a fully assembled haplotype prediction and *I*(*a *= *b*) represents the indicator function which assumes 1 if *a *= *b *and 0 otherwise. A simple modification of the above formula to the partially assembled haplotype segments might be

$$R^{\prime }\left(\Phi \right)=1-\frac{1}{2M}\overset{B}{\underset{b=1}{\sum }}\text{min}\left[D\left(\Phi_{b},\;\Phi_{b}^{\left(t\right)}\right),\;D\left(\Phi_{b},{\bar{\Phi }}_{b}^{\left(t\right)}\right)\right].$$

However, this definition is inconvenient because the minimization is applied for each segment and this accuracy measure can always be improved just by breaking a segment into smaller pieces at random positions.

**Figure 2:**
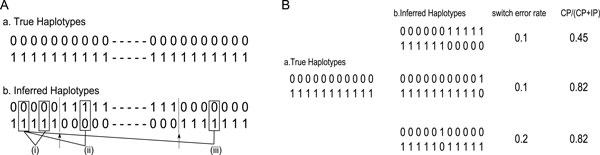
**An illustration of pair consistency**. Consistency of pair sites. A. a. We assume that the two true haplotypes are the sequences of all 0 and all 1. b. Inferred haplotypes contain switch errors indicated by the arrows: (i) a consistent pair, (ii) an inconsistent pair, and (iii) if there are an uncontrolled number of switch errors between a pair, the probabilities of being consistent or inconsistent are both 0.5. B. The example of the case that switch error rate is not suitable to evaluate the quality of the segment. The consistency of a reconstructed haplotype which has single switch error in the middle (top) is high than a reconstructed haplotype which has single switch error located at an end of the segment, but switch error rate cannot distinguish these situations. Two contiguous switch errors, which are caused by sequencing error or genotyping error and do not disrupt the consistency between front and back parts, are regarded as twice of a single switch error in switch error rate (bottom).

The switch error rate [13] is another measure used for comparing SIH algorithms. A switch error is defined by the inconsistency between Φ and Φ^(*t*) ^at neighboring heterozygous sites: $\left(\varphi_{j},\;\;\varphi_{j+1}\right)=\left(\varphi_{j}^{\left(t\right)},\;\;{\bar{\varphi }}_{j+1}^{\left(t\right)}\right)\;\text{or}\;\left({\bar{\varphi }}_{j}^{\left(t\right)},\;\;\varphi_{j+1}^{\left(t\right)}\right)$. The switch error rate is defined by the total number of switch errors divided by the total number of neighboring pairs of heterozygous sites in all the segments. Although the switch error rate is useful for comparing different algorithms, it does not reflect the global influence of switch errors. Figure 2(B) shows the example of the case that the switch error rate is not suitable to evaluate the quality of the segments. A single switch error in the middle of a reconstructed haplotype segment has a greater influence on downstream analyses such as detecting amplified haplotypes [23] than a switch error located at an end of the segment (top and middle of Figure 2(B)). Two contiguous switch errors, which are likely to be caused by sequencing error or genotyping error, do not disrupt the consistency between front and back parts of the haplotype segments. However, such two contiguous switch error disrupt twice in terms of switch error rate (bottom of Figure 2(B)).

Here, we propose another simple accuracy measure based on the pairwise consistency of the prediction with the true haplotypes. This pairwise consistency score is inspired by the *D'*-measure of linkage disequilibrium where the statistical correlations among population genomes are measured for pair sites. Similarly to the switch error, a pair of heterozygous sites *j *and *j*' (*j < j*') is defined as consistent if $\left(\varphi_{j},\;\varphi_{j^{\prime }}\right)=\left(\varphi_{j}^{\left(t\right)},\;\varphi_{j^{\prime }}^{\left(t\right)}\right)\;\text{or}\;\left({\bar{\varphi }}_{j}^{\left(t\right)},{\bar{\varphi }}_{j^{\prime }}^{\left(t\right)}\right)$, and inconsistent otherwise. A pair (*j*, *j*') in a haplotype segment is consistent if there is no switch error in range [*j, j^'^*] and inconsistent if there is one switch error in the segment. If there are uncontrolled number of switch errors in range [*j, j*'], the probabilities that pair (*j, j^'^*) is consistent or inconsistent are both 0.5, which is equivalent to selecting a random phase at each site (Figure 2(A)). For each haplotype segment, we count the consistent and inconsistent pairs. The total numbers of consistent and inconsistent pairs over all the haplotype segments are denoted by CP and IP, respectively. We define *precision *by CP/(CP + IP). This is used as the measure of accuracy in the later sections. Unlike the switch error rate, this precision accounts for the global influence of switch errors because a switch error in the middle of a haplotype segment leads to a much smaller CP than switch errors at an end of the segment.

We define the total prediction space as follows. We consider a graph whose nodes are the set of all the heterozygous sites. We connect two nodes by an edge if there is a fragment spanning both the sites. We collect all the connected components with at least two nodes and consider each of the corresponding clusters of heterozygous sites as an independent segment. The total number of pairs is the sum of the numbers of all the pair sites over the segments. Although it is rare, there are cases in which some segments consist of noncontiguous heterozygous sites. For example, segment sets such as {(1, 4, 5), (2, 3)} and {(1, 3), (2, 4, 5)} may occur for the consecutive heterozygous sites (1, 2, 3, 4, 5). We define *recall *as the ratio of the predicted pairs divided by the total number of pairs. Because the previous algorithms provide no score to limit the prediction to highly confident regions, recall is always nearly equal to one for these algorithms. On the other hand, our algorithm is able to make predictions with high precision at the expense of reduced recall.

A more detailed discussions of other accuracy measures is given in Additional file 1.

### Potential chimeric fragments

The processed sequence data derived from fosmid pool-based next-generation sequencing might contain chimeric fragments if a pool contains DNA fragments derived from the same region of different chromosomes and reads with different chromosomal origins are merged into a single SNP fragment. By using the trio-based haplotypes, we compute the 'chimerity' of each SNP fragment *f *by measuring the change of its likelihood after breaking it into two pieces:

$$\text{chimerity}\left(f\right)=-\text{log}\left(\frac{{\text{max}}_{h\in H}P_{0}\left(f|h\right)}{{\text{max}}_{j\in X\left(f\right),h\in H}P_{0}\left(f\leq_{j}|h\right)P_{0}\left(f{>}_{j}|\bar{h}\right)}\right),$$

$$P_{0}\left(f|h\right)={\left(1-\alpha_{0}\right)}^{n\left(f,h\right)}\alpha_{0}^{|X\left(f\right)|-n\left(f,h\right)},$$

where *n*(*f*, *h*) is the number of sites at which the fragment *f *matches with the true haplotype *h*, *f_≤j _*and *f_>j _*represent the left and right parts of fragment *f *divided at site *j*, and *α*_0 _= 0.028 is the empirical sequence error rate computed by comparing the true haplotypes and all the SNP fragments. We removed potential chimeric fragments with chimerity higher than a given threshold. We recomputed the accuracies for this removed dataset and compared them with those for the original dataset.

## Results and discussion

### Comparison of pairwise accuracies

**Figure 3:**
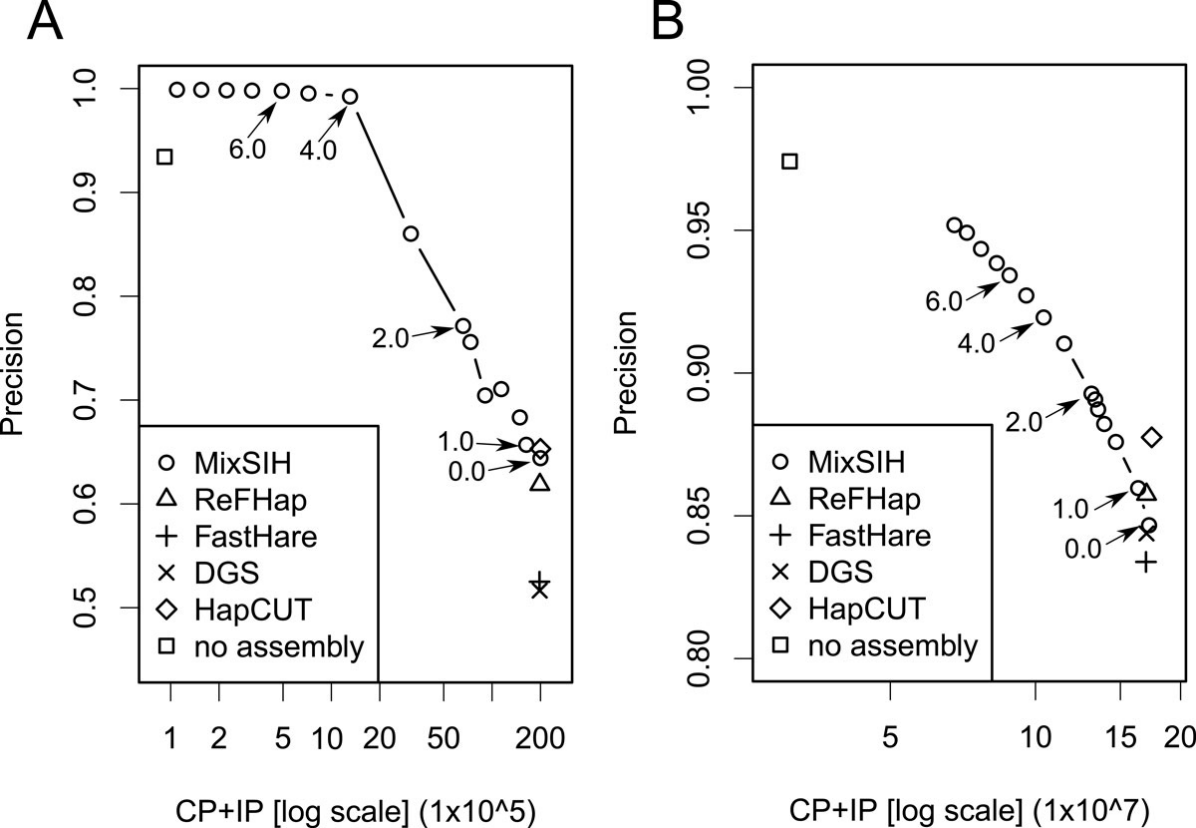
**Comparison of pairwise accuracies**. Precision curves based on the consistent pair counts. The *x*-axis represents the number of predicted pairs in log scale. The arrows indicate the MC thresholds. The accuracies are computed for the simulation dataset (A), and the real dataset (B): □ no assembly; ○ MixSIH; Δ ReFHap; + FastHare; × DGS. In the simulation, we set *M *= 2000 and repeated the experiment 10 times for each algorithm; average values are plotted.

We examined whether MixSIH can extract the accurate haplotypes regions by using MC. Figure 3 shows the accuracies derived from counting the consistent pairs. The *x*-axis is the number of predicted pairs (CP+IP) and the *y*-axis is the precision (CP/(CP+IP)). We have also shown the accuracy for the prediction without the haplotype assembly where the phase of each pair is determined by majority voting of spanning fragments. Figure 3A shows that the precisions of all the algorithms are around 0.5-0.6 at recall ~ 1.0, indicating that there are many switch errors in the predictions and the quality of assembled haplotypes are not much different from picking phases randomly. By increasing the MC threshold, the precision of MixSIH improves rapidly and becomes close to one around MC = 4 at recall 0.07. The recall of unassembled haplotypes is about 0.005 with precision 0.93, which is 20 times smaller than the recall 0.1 of MixSIH at the same precision. For the real dataset, the precision of the algorithms is around 0.85 at recall ~ 1.0, which is much higher than the precision for the simulation dataset. This is because there are many small fragment clusters for which the correct haplotypes are easily predicted. The accuracy of MixSIH can still be improved with precision up to 0.95 at the expense of deleting about 3/5 of weakly supported pairs from the prediction. However, it does not reach the precision of unassembled haplotype prediction. We discuss this issue in the next subsection.

### Effects of potential chimeric fragments

**Figure 4:**
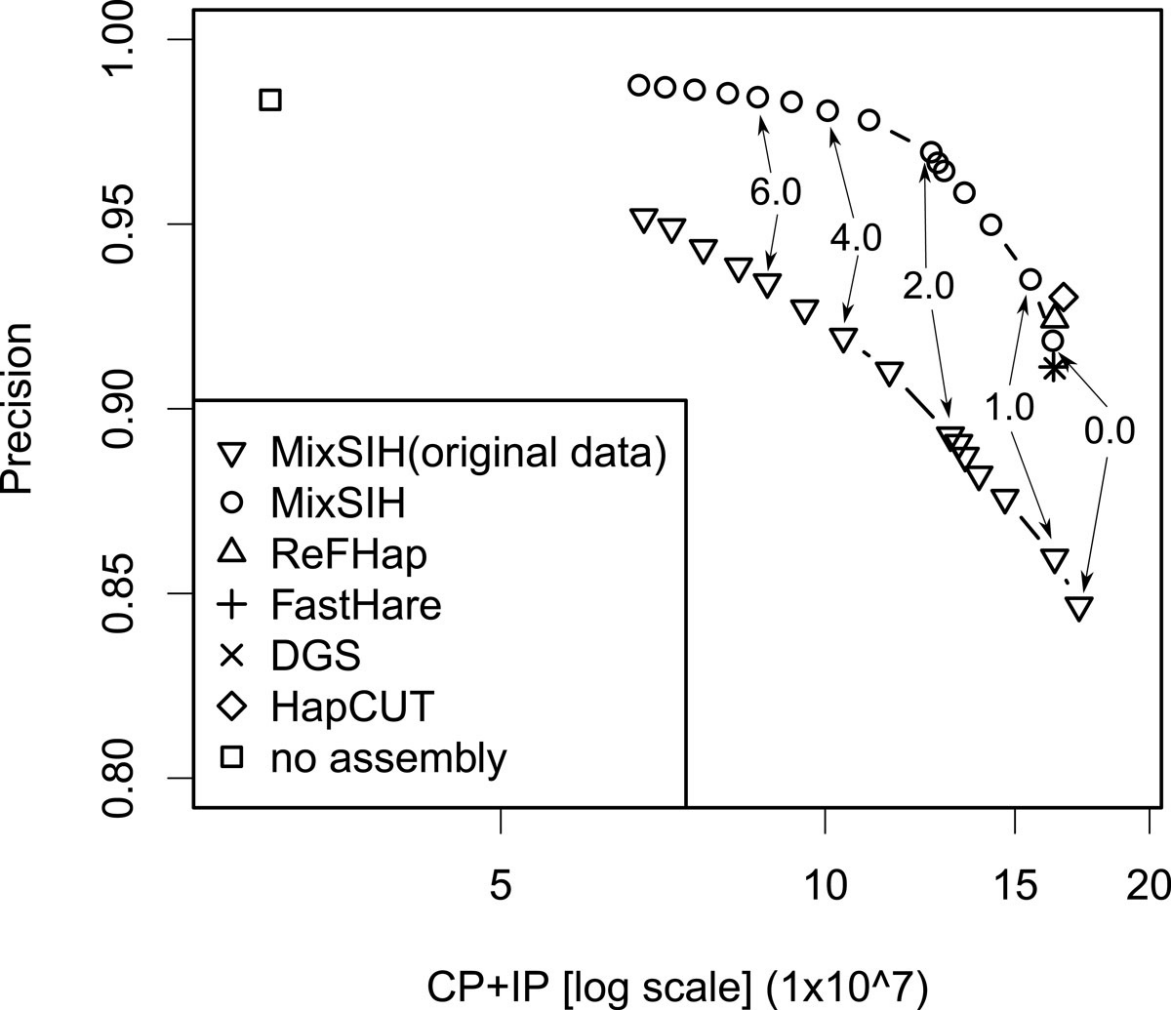
**Effects of potential chimeric fragments**. The precisions of the algorithms for the dataset in which fragments with chimerity greater than 10 are removed. For comparison, the precisions of MixSIH for the original dataset are also shown as diamonds.

Inspecting the switch errors in the prediction for the real dataset, we found that there are potential chimeric fragments that have a considerable effect on the pairwise accuracies. A chimeric fragment is defined as a fragment whose left and right parts match different chromosomes very well. Such fragments can occur in fosmid pool-based next-generation sequencing data. We show the chimerity distribution in Additional file 1. We computed the accuracy of MixSIH for a fragment dataset in which the fragments with chimerity higher than a given threshold are removed. We experimented with several chimerity thresholds and we found that the accuracy improves with decreasing chimerity thresholds and saturated at about chimerity threshold 10, which corresponded to the case that only 1.65% (4,482/271,184) of the fragments were removed. We show the accuracies for different chimerity thresholds in Additional file 1. We also show that the fragments whose chimerity is over 10 are indeed chimeric in Additional file 1. Figure 4 shows the precision curves for the dataset of removed fragments. The accuracies are considerably higher for this dataset, and the precision now reaches that of the unassembled prediction at recall 0.5 with MC threshold 6.0. We also show the effects of chimeric fragments on simulation data in Additional file 1.

These results suggest that more careful data processing to avoid spurious chimeric fragments is necessary to obtain high-quality haplotype assembly.

### Incorporation of the trio-based data

Although the trio-based statistical phasing method can determine most of the phases of the sites, there still exist SNP sites whose phases cannot be determined by this method. SIH is capable of determining the phases which are not determined by the trio-based data, and we can obtain more complete haplotypes data by combining both of the SIH-based data and the trio-based data. To examine how many phases of the sites can be determined anew by combining both of the SIH-based data and the trio-based data, we devise a method that combines both information to determine the phases (see the Additional file 1). By using this method, about 82% (237,950/291,466) of the phases of the sites which are undetermined by trio-based data could be determined anew and totally about 97% (1,601,381/1,654,897) of the phases could be determined by both the methods. This result suggests that almost all of the phases of the sites can be determined by using both of the SIH-based data and the trio-based data.

### Spatial distribution of MC values

**Figure 5:**
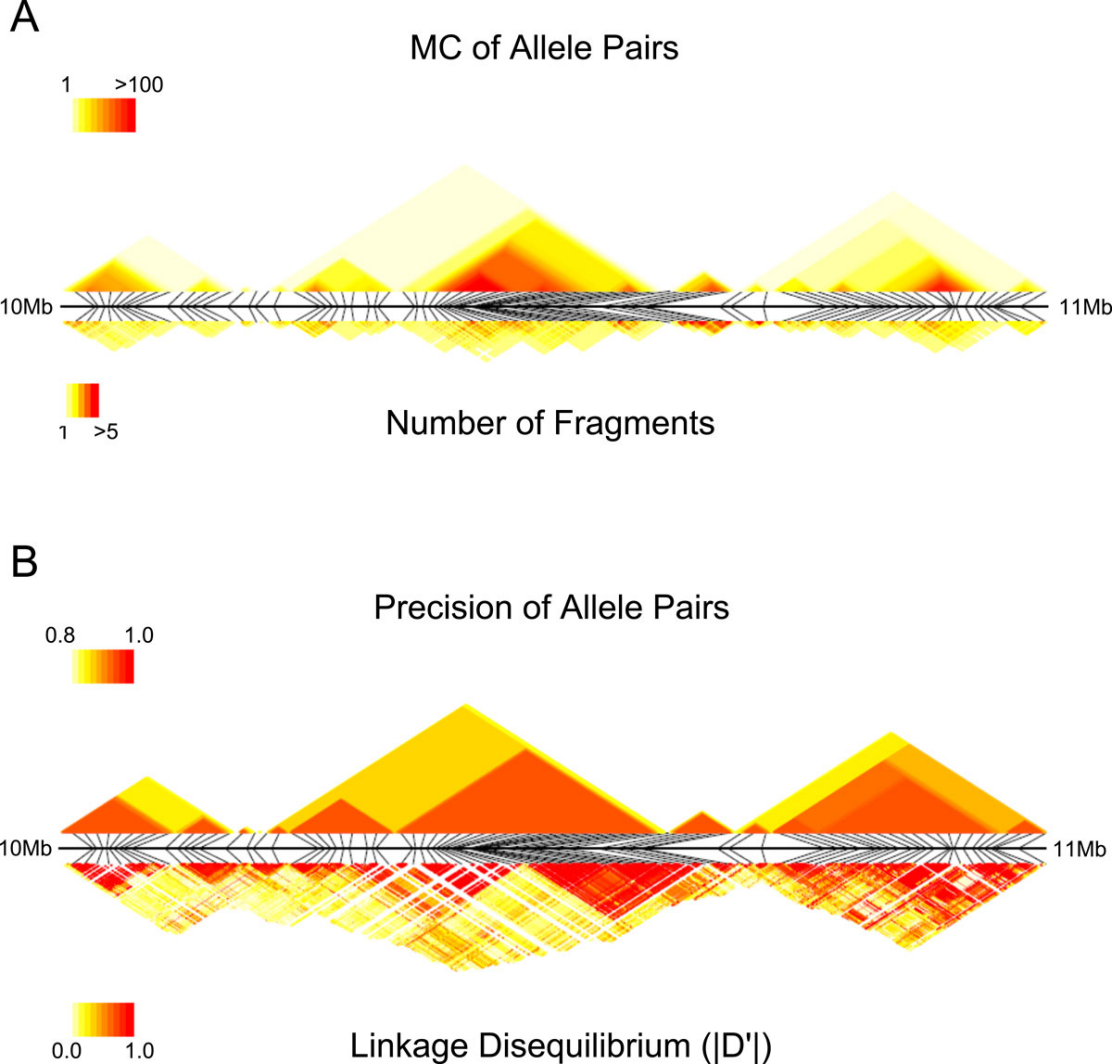
**Spatial distribution of MC and LD**. A. A colored density plot of the MC values and the number of fragments. The *x*-axis represents the co-ordinates of heterozygous sites. The actual locations of the sites in genome coordinates are shown by thin black diagonal lines and the black horizontal line represents a 10-11 megabase region of chromosome 20. The upper densities represent the connectivity values. The lower densities represent the number of fragments spanning the pair sites. B. A colored density plot of the precisions (upper) and the absolute normalized linkage disequilibrium *|D*'*| *(lower) for the same region.

Figure 5A shows an example of the spatial distribution of the MC values for the real dataset. The regions that are densely covered tend to have large MC values. On the other hand, the MC values are low in chromosomal regions with sparse heterozygous sites because few fragments span two or more sites. Figure 5B shows the density plot of MC values which are converted to the corresponding precisions using the graph of Figure 5B, and the absolute normalized linkage disequilibrium *|D*'*|*. SIH can accurately infer the haplotypes in many regions with low linkage disequilibrium, but there are also regions with reduced precision and high *|D*'*| *values. This suggests that the accuracy of predictions might be improved by using both pieces of information.

### Dependency of MC values on the fragment parameters

**Figure 6:**
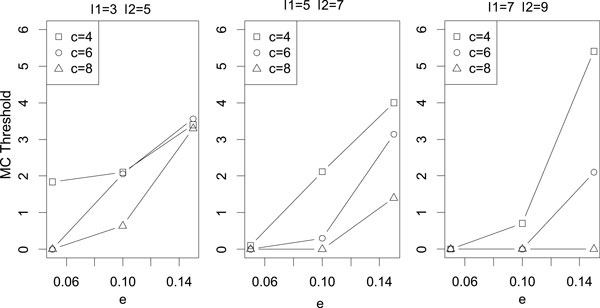
**Dependency of MC values**. Dependency of the lowest MC value with precision *≥ *0.95 for coverage *c*, fragment length [*l*_1_, *l*_2_], and error rate *e*. The experiments were repeated 10 times, and the average values are plotted.

Figure 6 shows the dependency of MC values on the quality of the input dataset. In these figures, the minimal MC threshold that achieves precision *≥ *0.95 (*y*-axis) is plotted for different fragment length ranges [*l*_1_, *l*_2_] (three panels), coverages *c *(three lines), and error rates *e *(*x*-axis). They show that the MC threshold must be increased to obtain high-quality assembly for low-coverage, highly erroneous data, while it has a minor dependence on the typical fragment length. However, the overall scale of the MC threshold changes relatively moderately and it is bounded above at MC = 6 for the tested cases. We also calculated the dependency of MC values on the input dataset which include chimeric fragments and the results were almost the same (see the Additional file 1). Hence we set the default MC threshold to 6.0 in our software.

### Optimality of inferred parameters

**Figure 7:**
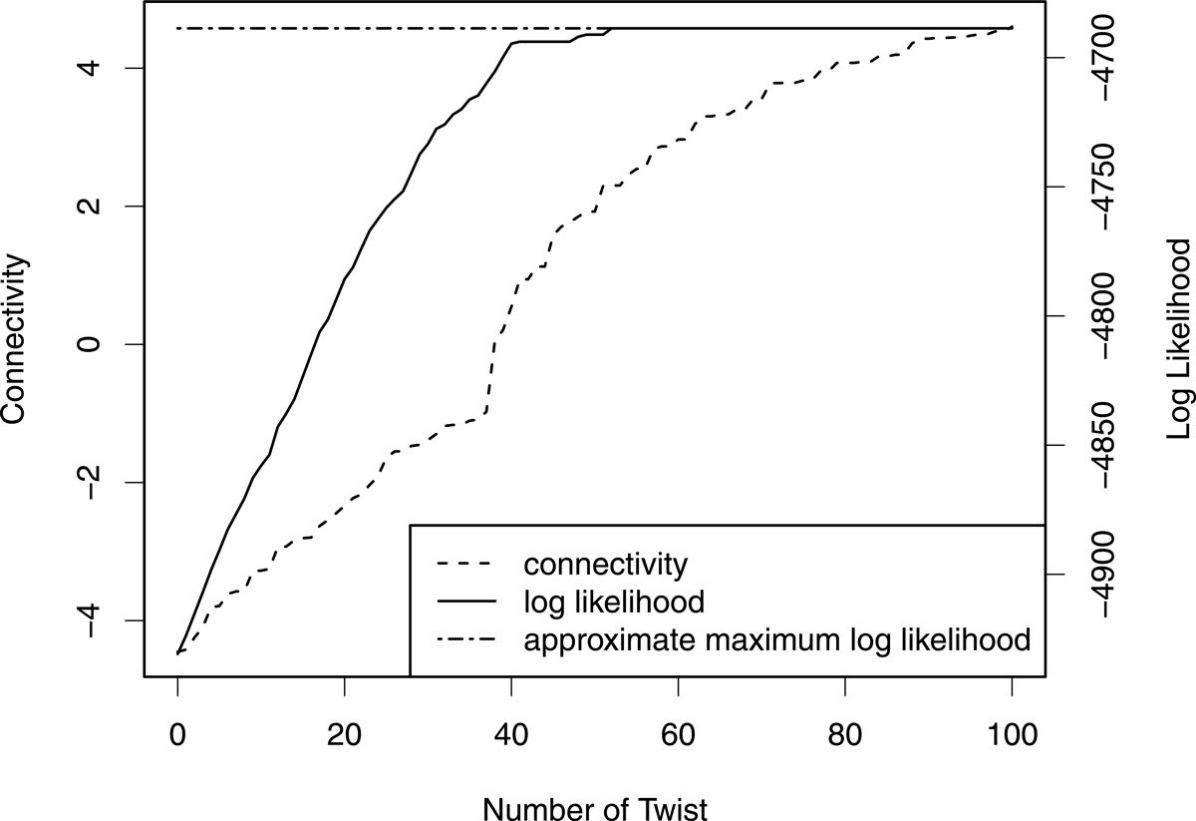
**Optimality of inferred parameters**. Increase of log likelihood values for each iteration. The dotted line represents the approximate maximal log likelihood; the solid line, the changes of the optimized log likelihood for each twist operation; the broken line, the connectivity values at the positions that the optimizing parameters are twisted.

We use a heuristic method for parameter optimization to avoid sub-optimal solutions. To test whether the optimized parameters actually reach the global optimum, we compared the log likelihood of the optimized parameters with the approximate maximal log likelihood obtained by optimizing the parameters with an initial condition in which the optimal solution falls into the set of true haplotypes; we add one to the Dirichlet parameters for the true phase probability: that is, $\lambda_{jv}=\lambda_{jv}^{\left(0\right)}+1\;\text{if}\;v=\varphi_{j}^{\left(t\right)}$and $\lambda_{jv}=\lambda_{jv}^{\left(0\right)}$ otherwise, where $\lambda_{jv}^{\left(0\right)}$ is hyperparameters of the Dirichlet distribution and $\varphi_{j}^{\left(t\right)}$ is the true phase at site *j*. Figure 7 shows the changes of the log likelihood for each twist operation. It also shows the connectivity values at the sites where the parameters Λ are twisted. The log likelihood increases monotonically and reaches the approximate maximal likelihood after 50 twist iterations. The connectivity values also increase monotonically in most cases. The figure implies that the parameters converge to the global optimum upon repeating the twist operation.

### Comparison of running times

**Figure 8:**
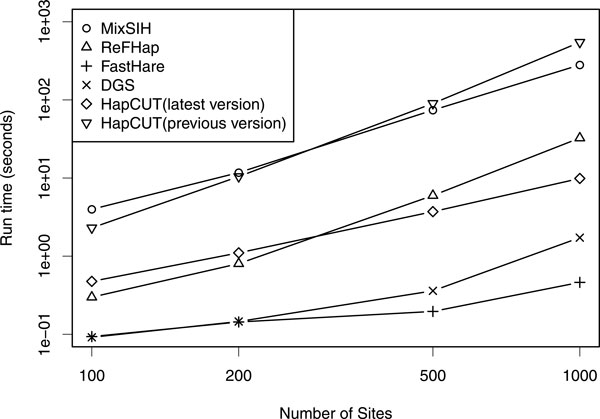
**Running times**. The running times of the tested algorithms. The *x*-axis is the number of sites. The *y*-axis is the running time in seconds. Both are displayed on a logarithmic scale.

Figure 8 shows the runtimes of the test programs. Bansal released the faster version of HapCUT recently, so we calculated the runtimes of both latest and previous version of HapCUT. Our method applies the VBEM algorithm repeatedly and hence is rather slow. It is comparative to HapCUT(previous versoin) and about 10-fold slower than both ReFHap and HapCUT(latest versoin), and from 50-fold to 500-fold slower than both FastHare and DGS. Considering that NA12878 has about 1.23 × 10^5 ^heterozygous sites on chromosome 1, it is roughly estimated that MixSIH takes about 15 days to finish haplotyping for the data whose connected component includes all heterozygous sites, and MixSIH is still manageable for such chromosome-wide data.

## Conclusions

With advances in sequencing technologies and experimental techniques, single individual haplotyping (SIH) has become increasingly appealing for haplotype determination in recent years. In this paper, we have developed a probabilistic model for SIH (MixSIH) and defined the minimal connectivity (MC) score that can be used for extracting accurately assembled haplotype segments. We have introduced a new accuracy measure, based on the pairwise consistency of the inferred haplotypes, which is intuitive and easy to calculate but nevertheless avoids some of the problems of existing accuracy measures. By using the MC scores our algorithm can extract highly accurate haplotype segments. We have also found evidence that there are a small number of chimeric fragments in an existing dataset from fosmid pool-based next-generation sequencing, and these fragments considerably reduce the quality of the assembled haplotypes. Therefore, a better data processing method is necessary to avoid creating chimeric fragments.

Our program uses only read fragment data derived from an individual. However, it is expected that more powerful analyses could be made by combining SIH algorithms with statistical haplotype phasing methods that use population genotype data. An interesting possibility would be to construct a unified probabilistic model that infers the haplotypes on the basis of both kinds of data.

## Abbreviations

SIH: Single Individual Haplotyping; MC: Minimum connectivity.

## Competing interests

The authors declare that they have no competing interests.

## Authors' contributions

HM designed the probabilistic model, implemented the software, performed the analyses, and wrote the paper. HK contributed to develop the model, designed the experiments and wrote the paper. Both authors read and approved the final manuscript.

## Supplementary Material

Additional file 1 **This file includes the explanation of our model, detail of the parameter optimization and some additional analyses**. Click here for file

## Declarations

The publication costs for this article were funded by a Grant-in-Aid for Young Scientists (21700330), and a Grant-in-Aid for Scientific Research (A) (22240031).

This article has been published as part of *BMC Genomics *Volume 14 Supplement 2, 2013: Selected articles from ISCB-Asia 2012. The full contents of the supplement are available online at http://www.biomedcentral.com/bmcgenomics/supplements/14/S2.
