# Supplementary material for: MixSIH: a mixture model for single individual haplotyping
Source: BMC Genomics. 2013 Feb 15;14(Suppl 2):S5. doi: 10.1186/1471-2164-14-S2-S5 (PMC3582441; doi:10.1186/1471-2164-14-S2-S5)
Supplement: Additional file 1 — This file includes the explanation of our model, detail of the parameter optimization and some additional analyses. [file 1471-2164-14-S2-S5-S1.pdf]

Supplementary Text for MixSIH: a mixture model for single individual haplotyping.

Hirotaka Matsumoto, Hisanori Kiryu

October 15, 2012

1 Difference Between Our Model and Existing Models

There are a number of differences between our model and those of [5] and [6]. Our model takes a 'mixture model' approach: each fragment is emitted independently of the other fragments and a partial phase vector $\Phi^{(i)} \in \Delta(f_i)$ is independently drawn for each fragment f_i:

$$P(F|\Theta) = \sum_{H \in \mathcal{H}^{\otimes N}} \prod_{i=1}^{N} \sum_{\Phi^{(i)} \in \Delta(f_i)} P(f_i|h_i, \Phi^{(i)}) p^m(h_i) P(\Phi^{(i)})$$

On the other hand, [5] and [6] take a 'hidden variables' approach: the model first draws a full-length phase vector Φ, then all the fragments are emitted from this common phase vector Φ:

$$P(F|\Theta) = \sum_{\Phi \in \Delta^{\otimes M}} P(\Phi) \sum_{H \in \mathcal{H}^{\otimes N}} \prod_{i=1}^{N} P(f_i|h_i, \Phi) p^m(h_i)$$

Although their model might look somewhat more natural, since the fragments are actually derived from the fixed true chromosomes, the computation of the likelihood function is quite costly; we need either to traverse all the $|\Delta|^M$-phase patterns (where $|\Delta|$ is the number of possible phases at each site), or to traverse all the $2^{|F^c(j)|}$-patterns for assigning haplotype origins $h_i \in \mathcal{H}$ to covering fragments $f_i \in F^c(j)$ for each site j. Therefore, it is impractical to use their model to compute a likelihood for genome-scale data. On the other hand, our model considers only one fragment at a time and the complexity of the likelihood computation is only $|\Delta| \times \sum_{i=1}^{N} |X(f_i)|$. Although our model loses some complicated correlations among fragments, it still takes into account the allele co-occurrences within each fragment.

2 Variational Bayes Expectation Maximization Algorithm

We set the prior probabilities for parameters Θ to be those of the Dirichlet distribution with hyperparameters $\Lambda^{(0)} = \{\lambda_{j\nu}^{(0)}\}$:

$$P(\Theta) = \prod_{j=1}^{M} \mathrm{Dir}\left(\theta_j|\lambda_j^{(0)}\right)$$
$$\mathrm{Dir}(\theta_j|\lambda_j^{(0)}) = Z\left(\lambda_j^{(0)}\right)^{-1} \prod_{\nu} (\theta_{j\nu})^{\lambda_{j\nu}^{(0)}} ,$$
$$Z\left(\lambda_j^{(0)}\right) = \left[\prod_\nu \Gamma\left(\lambda_{j\nu}^{(0)}\right)\right] / \Gamma\left(\sum_\nu \lambda_{j\nu}^{(0)}\right) ,$$

where $\Gamma(x)$ is the gamma function, and we set $\lambda_{j\nu}^{(0)} = 0.5$ for all j and ν.

The solutions for $Q^{H\Psi}(H,\Psi)$ and $Q^{\Theta}(\Theta)$ have the form

$$Q^{H\Psi}(H,\Psi) = \frac{1}{Z^{H\Psi}} \exp\left(\sum_{i=1}^{N} \sum_{h\in\mathcal{H}} \sum_{j\in X(f_i)} \sum_{\nu\in\Delta} \mathcal{I}_{ihj\nu} \log(\beta_{ihj\nu})\right) ,$$
$$Q^{\Theta}(\Theta) = \prod_{j=1}^{M} \mathrm{Dir}(\theta_j|\lambda_{j\nu}) ,$$

where $Z^{H\Psi}$ represents a normalization constant and $\beta_{ihj\nu}$ and $\lambda_{j\nu}$ are the hyperparameters that specify the posterior distributions. Because $Q^{H\Psi}(H,\Psi)$ and $Q^{\Theta}(\Theta)$ are dependent on each other through the dependencies among the hyperparameters, they cannot be found simultaneously. Therefore, we optimize $\beta_{ihj\nu}$ and $\lambda_{j\nu}$ by repeating two computational procedures, called VBE and VBM.

In the VBE step, we calculate the expectations

$$\gamma_{ihj\nu} = \sum_{H\Psi} \mathcal{I}_{ihj\nu} Q^{H\Psi}(H,\Psi) = \gamma_{ih}^{(1)} \gamma_{ihj\nu}^{(2)} ,$$
$$\gamma_{ih}^{(1)} = \frac{\prod_{j\in X(f_i)} (\sum_{\nu\in\Delta} \beta_{ihj\nu})}{\sum_{h'} \prod_{j\in X(f_i)} (\sum_{\nu\in\Delta} \beta_{ih'j\nu})} ,$$
$$\gamma_{ihj\nu}^{(2)} = \frac{\beta_{ihj\nu}}{\sum_{\nu'\in\Delta} \beta_{ihj\nu'}} .$$

In the VBM step, we update the Dirichlet parameters $\lambda_{j\nu}$ and then compute expectation $w_{j\nu}$ as well as $\beta_{ihj\nu}$:

$$\lambda_{j\nu} = \lambda_{j\nu}^{(0)} + \sum_{i=1}^{N} \sum_{h\in\mathcal{H}} \gamma_{ihj\nu} ,$$
$$w_{j\nu} = \int d\Theta \log(\theta_{j\nu}) Q^{\Theta}(\Theta) = \psi(\lambda_{j\nu}) - \psi\left(\sum_\nu \lambda_{j\nu}\right) ,$$
$$\beta_{ihj\nu} = p^e(f_{ij}|\nu_h) \exp(w_{j\nu}) .$$

3 Iterative Twist Operations to Avoid Sub-optimal Solutions

We optimize the parameters as follows.

1. Set $\lambda_{k\nu}^{(0)} = 0.5$ for all k and ν and initialize Λ with $\lambda_{k\nu} = \lambda_{k\nu}^{(0)} + r_{k\nu}$. Here, $r_{k\nu}$ are random numbers sampled from the uniform distribution in the range $[0.0, 0.1]$. They are necessary to avoid the symmetric point of the likelihood function. Let S be the empty set, and set score $= -\infty$ and $\Lambda_1 = \Lambda$.

2. Do variational Bayes expectation maximization [1] with initial parameter Λ_1 until the parameters converge or the number of iterations exceeds a given limit (100). Let score$'$ and Λ' denote the converged likelihood and converged parameter set, respectively.

3. If score $<$ score$'$ then set score $=$ score$'$, $\Lambda = \Lambda'$.

4. Select the site j out of sites $X \setminus S$ that has the smallest connectivity c_j with respect to the model Λ.

5. Add j to S if j has already been selected once in the previous iterations.

6. Set $\Lambda_1 = \Lambda$ and twist Λ_1 at site j. (The concept of 'twisting' is described in 'The Minimum Connectivity Score' subsection in the main paper.)

7. If $c_j > 7.0$ or $X = S$, then terminate, otherwise go to step 2.

4 Dependency of the parameter α and the coverage

We examined the influence of α on the efficiency of MixSIH and calculated the switch error rates of MiSIH with different α on the simulation data whose connected component include all sites. We also calculated the switch error rates of ReFHap, FastHare, DGS and HapCUT. Table 1 shows a comparison of the switch error rates. It shows that the scores of MixSIH whose error rate parameter α are set to sequence error rates e tend to be low, especially when sequence error rates are high. It also shows that MixSIH outperforms FastHare and DGS, and is comparable to ReFHap and HapCUT under the condition that the inferred haplotypes are completely connected. This data also show that almost complete haplotype information is obtained when the coverage are high.

5 Comparison of Accuracy Measures

Because of the equivalence of predictions between the switched haplotypes as explained above, measuring the difference between $\Phi^{(t)}$ and Φ is nontrivial.

Table 1: Comparison of switch error rates (%) for simulated data with varying coverage c and sequence error rates e. We set $M = 100$, and repeated the evaluation 100 times for each parameter; average values are shown. The parameter α is the error rate parameter of the MixSIH model. The best scores are shown in bold for each (e, c) pair.

	$e = 0.0$	$e = 0.025$	$e = 0.05$			$e = 0.075$			$e = 0.1$		
	$c = 3$	$c = 3$	$c = 3$	$c = 5$	$c = 8$	$c = 3$	$c = 5$	$c = 8$	$c = 3$	$c = 5$	$c = 8$
MixSIH(α=0.00)	**0.00**	0.03	0.33	**0.02**	0.01	0.79	0.10	**0.00**	1.90	0.36	**0.01**
MixSIH(α=0.05)	**0.00**	0.03	0.31	**0.02**	**0.00**	0.84	0.08	**0.00**	1.81	0.24	**0.01**
MixSIH(α=0.10)	**0.00**	0.04	**0.29**	0.03	**0.00**	0.76	0.08	**0.00**	1.80	**0.18**	**0.01**
ReFHap	**0.00**	**0.01**	**0.29**	0.03	**0.00**	**0.75**	0.10	**0.00**	1.88	0.27	**0.01**
FastHare	**0.00**	0.15	0.63	0.09	0.02	1.19	0.31	0.06	2.47	0.70	0.08
DGS	**0.00**	0.19	1.00	0.43	0.38	2.45	1.22	0.82	4.00	2.08	1.73
HapCUT	**0.00**	0.02	0.31	0.03	**0.00**	0.83	**0.06**	**0.00**	**1.67**	0.20	0.02

Many previous papers used the Hamming distance to measure the quality of assembled haplotypes [3]:

$$R(\Phi) = 1 - \frac{1}{2M} \min \left[D(\Phi, \Phi^{(t)}), D(\Phi, \bar{\Phi}^{(t)}) \right] ,$$

$$D(\Phi, \Phi') = \sum_{j=1}^{M} \sum_{h \in \mathcal{H}} I(\varphi_{jh} = \varphi'_{jh}) ,$$

where $I(a = b)$ represents the indicator function which assumes 1 if $a = b$ and 0 otherwise. This definition is not appropriate when we consider the accuracy of multiple, partially resolved haplotype segments. For example, there is no way for the SIH algorithms to relate the haplotypes of chromosome 1 to those of chromosome 2 because there is no fragment that overlaps with both the chromosomes. It is also impossible for any SIH algorithm to relate the haplotypes of two consecutive regions if there is no fragment that overlaps with both regions. Furthermore, we wish to extract confidently assembled sub-regions using the minimum connectivity thresholds. Therefore, it is desirable for the accuracy measures to allow comparisons on the set of partially assembled haplotype segments.

We now consider a simple extension of the Hamming distance measure. Let $\Phi = (\Phi_1, \Phi_2, \ldots, \Phi_B)$ be the set of partially assembled haplotype segments with M total sites, then a simple modification of the above formula might be

$$R'(\Phi) = 1 - \frac{1}{2M} \sum_{b=1}^{B} \min \left[D(\Phi_b, \Phi_b^{(t)}), D(\Phi_b, \bar{\Phi}_b^{(t)}) \right] .$$

However, this definition is inconvenient because the minimization is applied for each segment and this accuracy measure can always be improved just by breaking a segment into smaller pieces at random positions.

The switch error rate [2] is another measure used for comparing SIH algorithms. A switch error is defined by the inconsistency between Φ and $\Phi^{(t)}$ at neighboring heterozygous sites: $(\varphi_j, \varphi_{j+1}) = (\varphi_j^{(t)}, \bar{\varphi}_{j+1}^{(t)})$ or $(\bar{\varphi}_j^{(t)}, \varphi_{j+1}^{(t)})$. The

switch error rate is defined by the total number of switch errors divided by the total number of neighboring pairs of heterozygous sites in all the segments. Although the switch error rate is useful for comparing different algorithms, it does not reflect the global influence of switch errors. For example, a single switch error in the middle of a reconstructed haplotype segment has a greater influence on downstream analyses, through incorrect prediction of allele co-occurrences, than a switch error located at an end of the segment.

There are other measures, such as the minimum number of entries to correct (MEC) [4], the adjusted N50 (AN50) and its variants S50, N50 [7], and the quality adjusted N50 (QAN50). Apart from QAN50, these measures do not use the true haplotypes and there is no guarantee that the correct haplotypes have a higher score than incorrect ones. The procedure to compute the QAN50 score is complex and can be roughly described as follows. First the prediction is broken into smaller segments that do not contain any switch errors. For each segment an adjusted length score, which is the segment length in the reference genome multiplied by the proportion of heterozygous sites inside of the segment, is assigned. The segments are sorted in order of decreasing adjusted length scores and AN50 is defined as the threshold score such that half of heterozygous sites are covered by segments with scores greater than the threshold. Although this measure accounts for both the quality and segment sizes of the reconstruction, the complex interactions between inhomogeneity of the SNP density and fragment coverage seem to make it difficult to understand the practical utility of SIH algorithms by using their QAN50 scores.

In comparison to the switch error rate, which cannot account for genotyping errors in homozygous sites, the pairwise consistency score works without modification in the cases where homozygous sites are included in the prediction space. Furthermore, although the notion of pairwise consistency is applicable to haplotype segments that are not made up of simple contiguous sites, the definition of a switch error for such segments is somewhat ambiguous.

6 Potential Chimeric Fragments

Figure 1 shows the chimerity distribution of real data [2], which indicates that only a small proportion of the data has high chimerity. Figure 2 shows the accuracies for different chimerity thresholds, which suggests that the improvement of the accuracies saturates at around chimerity threshold 10.

The fragments whose chimerity were over 10 might be not indeed chimeric and these chimerity might be over 10 only by sequencing errors. To examine whether the fragments whose chimerity were over 10 were indeed chimeric or not, we calculated the probability that the chimerity of a fragment was over 10 only by sequencing errors. In the case that the fragment length was 18 which was about the average fragment length of the real data, the probability was about $4.8 \times 10^{-}5$ and we concluded threshold 10 was enough to consider a fragment as chimeric.

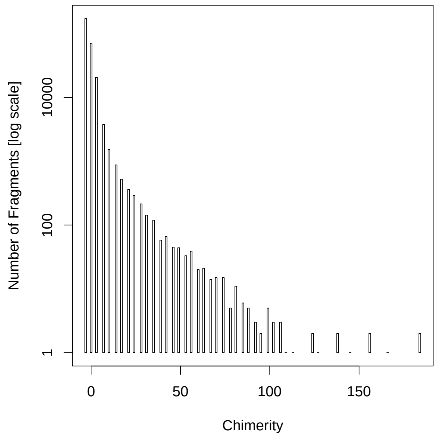

Figure 1: Chimerity distribution of the real dataset [2].

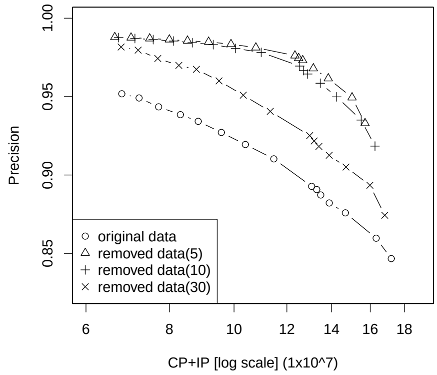

Figure 2: The precisions for the original dataset (○) and the datasets in which the fragments with chimerity greater than 5 (△), 10 (+), and 30 (×) are removed.

7 Incorporation of the trio data

We devise a method that combines both of the trio data and the fosmid data. The procedure is as follows:

1. Calculate chimerity of each fragment of the fosmid data by using the trio data, and remove the fragments whose chimerity is over 10.
2. Infer the haplotypes from the SNP fragments data in which the fragments with chimerity higher than 10 are removed.
3. Count the number of sites whose phases could be determined anew. The phase at site i is defined to be determined anew if it satisfies the following conditions.
   (a) The phase of the site i is not determined by trio-based method.
   (b) There exists a site j such that the phase of the site j is determined by the trio-based data and the MC between i and j (MC(j, i) or MC$(i+1, j)$) is higher than 6.

By using this method, about 82% (237,950/291,466) of the phases of the sites which are undetermined by the trio-based data could be determined anew and totally about 97% (1,601,381/1,654,897) of the phases could be determined by both the methods.

8 Simulation with Chimeric Fragments

To examine the influence of chimeric fragments for various analyses, we repeated the same analyses for simulation data which include chimeric fragments.

8.1 Comparison of Pairwise Accuracies for the Simulation Data with Chimeric Fragments

We examined the accuracy for the simulation data which included 1.5% of chimeric fragments, which was almost the same rate of the real data. Figure 3 shows the accuracies for the simulation data which include and don't include chimeric fragments, respectively. The precision of MixSIH for simulation data with chimeric fragments is lower than that for data without chimeric fragments around MC=1. The recall of MixSIH for data with chimeric fragments is almost same to the recall of MixSIH for data without chimeric fragments at MC=4 at which the precision becomes closed to one. This show that the influence of chimeric fragments is ignored with high MC threshold in this simulation data.

8.2 Dependency of MC Values on the Simulation Data with Chimeric Fragments

Figure 3 shows the dependency of MC values on the quality of the input dataset which include about 1.5% of chimeric fragments, which is almost the same rate

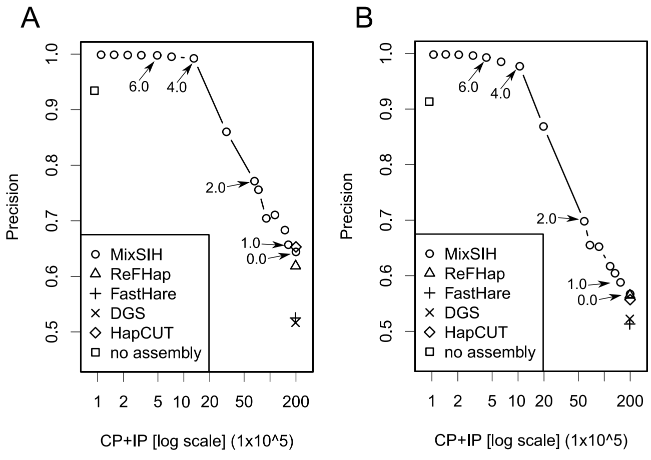

Figure 3: Precision curves based on the consistent pair counts. The x-axis represents the number of predicted pairs in log scale. The arrows indicate the MC thresholds. The accuracies are computed for the simulation dataset without chimeric fragments(A), and the simulation dataset with chimeric fragments (B): $\square$ no assembly; $\circ$ MixSIH; $\triangle$ ReFHap; $+$ FastHare; $\times$ DGS. In the simulation, we set $M = 2000$ and repeated the experiment 10 times for each algorithm; average values are plotted.

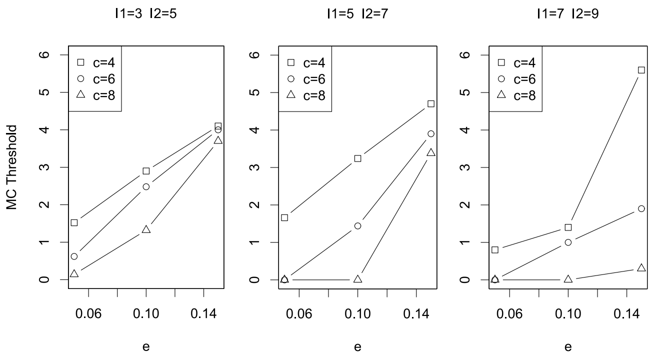

Figure 4: Dependency of the lowest MC value with precision ≥ 0.95 for coverage c, fragment length $[l_1, l_2]$, error rate e, including 1.5% of chimeric fragments. The experiments were repeated 10 times, and the average values are plotted.

of the real data. Most of the minimal MC thresholds for this input dataset are almost the same to those for dataset which don't contain chimeric fragments, but MC=6 is still enough strict to extract reliable haplotype regions.

References

[1] H. Attias. Inferring parameters and structure of latent variable models by variational Bayes. In *UAI'99*, pages 21–30, 1999.

[2] J. Duitama, G. K. McEwen, T. Huebsch, S. Palczewski, S. Schulz, K. Verstrepen, E. K. Suk, and M. R. Hoehe. Fosmid-based whole genome haplotyping of a HapMap trio child: evaluation of Single Individual Haplotyping techniques. *Nucleic Acids Res.*, 40:2041–2053, Mar 2012.

[3] F. Geraci. A comparison of several algorithms for the single individual SNP haplotyping reconstruction problem. *Bioinformatics*, 26:2217–2225, Sep 2010.

[4] D. He, A. Choi, K. Pipatsrisawat, A. Darwiche, and E. Eskin. Optimal algorithms for haplotype assembly from whole-genome sequence data. *Bioinformatics*, 26:i183–190, Jun 2010.

[5] J. H. Kim, M. S. Waterman, and L. M. Li. Diploid genome reconstruction of Ciona intestinalis and comparative analysis with Ciona savignyi. *Genome Res.*, 17:1101–1110, Jul 2007.

[6] L. M. Li, J. H. Kim, and M. S. Waterman. Haplotype reconstruction from SNP alignment. *J. Comput. Biol.*, 11:505–516, 2004.

[7] C. Lo, A. Bashir, V. Bansal, and V. Bafna. Strobe sequence design for haplotype assembly. *BMC Bioinformatics*, 12 Suppl 1:S24, 2011.
